# A phase III study to access the safety and efficacy of prolgolimab 250 mg fixed dose administered every 3 weeks versus prolgolimab 1 mg/kg every 2 weeks in patients with metastatic melanoma (FLAT)

**DOI:** 10.3389/fonc.2024.1385685

**Published:** 2024-09-04

**Authors:** Lev Demidov, Galina Kharkevich, Natalia Petenko, Vladimir Moiseenko, Svetlana Protsenko, Tatiana Semiglazova, Anastasia Zimina, Nadezhda Kovalenko, Natalia Fadeeva, Dmitry Kirtbaya, Igor Belogortsev, Denis Tantsyrev, Svetlana Odintsova, Alfia Nesterova, Karina Vorontsova, Yulia Makarycheva, Yulia Linkova, Arina Zinkina-Orikhan, Anna Siliutina, Irina Sorokina, Daria Liaptseva, Vladimir Chistyakov, Anton Lutsky

**Affiliations:** ^1^ FSBI “N.N. Blokhin National Medical Research Center of Oncology”, Ministry of Health (MoH) of the Russian Federation, Moscow, Russia; ^2^ State Budgetary Healthcare Institution (SBHI) “St. Petersburg Clinical Scientific and Practical Center for Specialized Types of Medical Care (Oncology)”, Saint Petersburg, Russia; ^3^ FSBI “N.N. Petrov National Medical Research Center of Oncology”, Ministry of Health (MoH) of the Russian Federation, Saint Petersburg, Russia; ^4^ Budgetary Healthcare Institution (BHI) of the Omsk Region “Clinical Oncology Dispensary”, Omsk, Russia; ^5^ State Budgetary Healthcare Institution (SBHI) “Volgograd Regional Clinical Oncology Dispensary”, Volgograd, Russia; ^6^ State Autonomous Institution of Healthcare (SAHI) “Chelyabinsk Regional Clinical Center of Oncology and Nuclear Medicine”, Chelyabinsk, Russia; ^7^ State Budgetary Healthcare Institution (SBHI) “Oncological Dispensary No. 2”, Ministry of Health (MoH) of the Krasnodar Region, Krasnodar, Russia; ^8^ Oncology Department, State Budgetary Healthcare Institution (SBHI) Leningrad Regional Clinical Hospital, Saint Petersburg, Russia; ^9^ Regional State Budgetary Healthcare Institution (SBHI) “Altai Regional Oncology Center”, Barnaul, Russia; ^10^ Oncology Department, Joint-Stock Company “Modern Medical Technologies”, Saint Petersburg, Russia; ^11^ State Autonomous Institution of Healthcare (SAHI) “Professor M.Z. Sigal Republican Clinical Oncology Dispensary of the Ministry of Health of the Republic of Tatarstan”, Kazan, Russia; ^12^ Moscow State Budgetary Healthcare Institution “A.S. Loginov MCSC of the Moscow City Healthcare Department”, Moscow, Russia; ^13^ State Budgetary Healthcare Institution (SBHI) “Samara Regional Clinical Oncology Dispensary”, Samara, Russia; ^14^ Clinical Research Department, Joint-Stock Company (JSC) Biocad, Saint Petersburg, Russia; ^15^ Oncology Department, Joint-Stock Company (JSC) Biocad, Saint Petersburg, Russia

**Keywords:** melanoma, PD-1 inhibitor, fixed dose, prolgolimab, immunotherapy

## Abstract

**Background:**

Prolgolimab is the first Russian PD-1 inhibitor approved for the first-line treatment of unresectable or metastatic melanoma and advanced non-small cell lung cancer. It was approved in two weight-based regimens of 1 mg/kg Q2W and 3 mg/kg Q3W, but because of re-evaluation of weight-based dosing paradigm, studying of a fixed-dose regimen was considered perspective.

**Methods:**

We conducted a multicenter, single-arm, open-label efficacy, pharmacokinetics, and safety study to obtain data that would allow the approval of the new flat dosing regimen of prolgolimab in patients with previously untreated unresectable or metastatic melanoma (BCD-100-8/FLAT, NCT05783882). The primary objective was to prove the non-inferiority of prolgolimab 250 mg Q3W versus prolgolimab 1 mg/kg Q2W for the treatment of patients with unresectable or metastatic melanoma in terms of ORR according to RECIST 1.1. Patients from the MIRACULUM study (BCD-100-2/MIRACULUM, NCT03269565) comprised a historical control group.

**Results:**

One hundred fourteen patients received prolgolimab 250 mg Q3W, and 61 patients received prolgolimab (Prolgo) 1 mg/kg Q2W (historical control). Objective response was achieved by 33.3% [95% confidence interval (CI): 24.8, 42.8] of patients in the Prolgo 250 mg group compared with 32.8% (95% CI: 21.3, 46.0) of patients in the Prolgo 1 mg/kg group. Risk difference was 0.00, 95% CI (−0.12; NA), *p* = 0.0082. Both regimens were well tolerated, and safety profiles were comparable. The pharmacokinetic analysis (PK) showed that the regimen with the fixed dose of 250 mg Q3W was characterized by higher PK parameters. The immunogenicity study did not detect binding antibodies to prolgolimab in any of the subjects.

**Conclusion:**

The obtained results showed that the selected fixed dosing regimen of prolgolimab 250 mg Q3W is characterized by efficacy and safety parameters comparable to that observed for the 1 mg/kg Q2W regimen.

## Introduction

The development of immune checkpoint inhibitors (ICIs) has fundamentally revolutionized the therapeutic landscape of cancer treatment modalities and has led to significant improvements in survival outcomes in patients with many tumor types, providing deep and durable responses.

Currently, ICIs of isotype G immunoglobulins (IgGs) are routinely used in clinical practice (prolgolimab, nivolumab, pembrolizumab, tislezumab, etc.). Clinical studies of currently available ICIs (except for ipilimumab) have not identified dose-limiting toxicity or dose-related efficacy, which tends to use doses that are well above the minimum effective doses (1). Their distribution volume is close to plasma volume, with limited tissue distribution. Metabolism and excretion of ICIs are not affected by renal and hepatic function due to the high molecular weight of ICIs and the absence of CYP enzyme involvement in their metabolism. Nonspecific (FcRn-mediated) pathway is predominant for ICIs, which explains the long half-life of these compounds and slow clearance. In addition, several studies have described exposure–response relationships where the clearance of ICIs was dependent on different covariates including sex, body weight, tumor burden, serum albumin levels, immunogenicity, and others (2–5). Currently available ICIs are immunoglobulins of isotype G (IgG), exhibiting similar pharmacokinetic properties. ICIs do not have a dose-dependent effect in the traditional point of view since the saturation of PD-1 receptors occurs at low concentrations of the drug (6, 7). This is explained by the high affinity between the drug and the PD-1 receptor. Therefore, the effectiveness of the PD-1 inhibitors does not depend on the dose, but it is controlled by biological factors and the effect on the immune system.

In 2020, the first Russian original PD-1 inhibitor prolgolimab (Forteca®, JSC Biocad, Saint Petersburg, Russia) was approved for the treatment of metastatic or unresectable melanoma based on the results of the phase II/III randomized clinical study MIRACULUM (NCT03269565) in the Russian Federation. Currently, the indications for the prolgolimab use are expanding. In the Russian Federation, at the end of 2023, prolgolimab in combination with platinum-based chemotherapy was approved as a first-line treatment of patients with advanced non-squamous NSCLC based on the phase III DOMAJOR study (NCT03912389) (8). There are two phase III clinical studies of prolgolimab currently underway: a study of the efficacy and safety of prolgolimab in combination with a platinum-based chemotherapy with and without bevacizumab in patients with advanced cervical cancer as a first-line treatment (FERMATA, NCT03912415), and a study of the sequential use of prolgolimab after neoadjuvant therapy of nurulimab + prolgolimab in high risk of recurrence melanoma patients with complete or near compete pathological response determined by the index lymph node (NEOMIMAJOR, NCT05751928).

Prolgolimab is a recombinant monoclonal antibody of IgG1 with a modified Fc fragment. The presence of the “LALA” mutation (Leu234Ala/Leu235Ala) in the Fc fragment of prolgolimab (amino acid substitution of two amino acids leucine with alanine) minimizes the effector properties of the antibody. Thus, prolgolimab does not bind to the FcγR receptors of macrophages, which heighten protection of the activated T lymphocytes population from possible antibody-dependent phagocytosis by macrophages and thereby enhance the antitumor effect. The unique binding epitope of prolgolimab ensures high saturation of PD-1 receptors at minimal concentrations. In the phase II part of the MIRACULUM study, an objective response rate (per irRECIST) was achieved by 38.1% of the patients with metastatic melanoma treated with prolgolimab monotherapy, a 24-month PFS—by 33.3% and a 24-month OS—by 57.1% of the patients. Associated with therapy all grades adverse events (AEs) were recorded in 55.6% of the patients, grades 3–4 severity—in 12.7%, serious AEs, and AEs that led to discontinuation of therapy—in 3.2% and 3.2% patients, respectively. The efficacy of prolgolimab is comparable to other drugs in the PD-1 inhibitor class, and the safety profile was most favorable in an indirect comparison (9, 10).

Initially, prolgolimab, as well as other anti–PD-1 drugs (nivolumab, pembrolizumab, tislezumab, etc.), was studied and approved in weight-based dosing: 1 mg/kg Q2W (9, 11) for patients with metastatic melanoma and 3 mg/kg Q3W for patients with advanced non-squamous NSCLC. Phase I study results showed that the maximum-tolerated dose (MTD) of prolgolimab was not reached and that there is no dose-response relationship, which aligns with other PD-1 inhibitors (12). Taking into account the biological mechanism of action of ICIs (1), the specific properties of mAbs (selective mechanism of action, wide therapeutic index) (2) (13), benefits of fixed dosing (increased convenience, elimination of wastage, increased safety resulting from reduced dosing errors, and improved dosing compliance) (3), accumulated data on other anti–PD-1 drugs (4) (14–17), the results of exposure-efficacy, exposure-safety, and pharmacokinetic (PK) studies of prolgolimab in early trials (5) (9, 12), we conducted a multicenter, single-arm, open-label efficacy, pharmacokinetics, and safety study to demonstrate non-inferiority of prolgolimab 250 mg every 3 weeks (Q3W) versus historical data for prolgolimab 1 mg/kg every 2 weeks (Q2W) in patients with unresectable or metastatic melanoma, as well as collecting pharmacokinetics and safety data (BCD-100-8/FLAT, NCT05783882).

## Materials and methods

### Study design and treatment

BCD-100-8/FLAT (NCT05783882) was a multicenter open-label phase III study of efficacy, pharmacokinetics and safety of prolgolimab (Prolgo) flat dosing regimen (250 mg Q3W) in patients with unresectable or metastatic melanoma.

The Q3W flat dose was selected based on population PK modeling along with efficacy and safety data on weight-based regimens from the BCD-100-2/MIRACULUM study (NCT03269565). The population PK analysis included patients receiving Prolgo 1 mg/kg Q2W (*N* = 63) and 3 mg/kg Q3W (*N* = 61) in the main (phase 2) part of the MIRACULUM study. A 2-compartment model was used to describe concentration-time data. The analysis of the population PK showed that 250 mg Q3W has predicted C_max_, C_av_ and C_trough_ after the 1st and the 10th infusions comparable to that observed for 3 mg/kg Q3W but higher than that observed for 1 mg/kg Q2W. Efficacy modeling and the results of the completed MIRACULUM study demonstrated that there is no dose-response relationship. Based on the above, it was expected that Prolgo 250 mg Q3W dosing regimen would be as effective as the approved 1 mg/kg Q2W regimen.

In the FLAT study eligible patients (aged ≥18 years) had unresectable or metastatic, previously untreated melanoma. Other main eligibility criteria included availability of tumor sample for immunohistochemical testing before starting neoadjuvant treatment, Eastern Cooperative Oncology Group (ECOG) performance status score 0 or 1, at least 1 measurable target lesion according to Response Evaluation Criteria In Solid Tumors (RECIST) 1.1 criteria confirmed by an independent reviewer and provided written informed consent. Patients were ineligible if they had received any prior treatment for unresectable or metastatic disease or prior anti-CTLA4 and/or anti–PD-1/PDL1/PD-L2 or targeted therapy.

Patients received intravenous Prolgo 250 mg Q3W until the development of intolerable toxicities or disease progression (whichever occurred first). At week 25 of therapy patients without progression were offered to participate in an extension clinical study, in which they continued to receive Prolgo 250 mg Q3W until the development of intolerable toxicity or disease progression.

Patients from the MIRACULUM study comprised a historical control group. Efficacy and safety data of the flat dosing regimen (250 mg Q3W) were compared with previously unpublished data obtained with the weight-based regimen 1 mg/kg Q2W in the confirmatory (phase 3) part of the MIRACULUM study. PK data obtained with flat dosing regimen were compared with previously unpublished data obtained with two weight-based dosing regimens in the main (phase 2) part of the MIRACULUM study (1 mg/kg Q2W and 3 mg/kg Q3W).

The FLAT study was conducted under the same conditions as the confirmatory part of the MIRACULUM study. Population-specific subject eligibility criteria, study centers, efficacy and safety assessment procedures, permitted prior and concomitant therapy of the primary disease were identical for both studies.

The primary endpoint was overall response rate (ORR) according to RECIST 1.1 criteria for 25 weeks of therapy. Secondary endpoints included ORR according to irRECIST criteria, disease control rate (DCR), time to response (TTR), and duration of response (DoR). DCR, TTR, and DoR were assessed per RECIST 1.1 and irRECIST criteria.

The safety endpoints were proportion of subjects with AEs of any grade, severe AEs (grade ≥3), serious AEs, AEs of any grade related to the study drug, immune-related AEs of any grade, severe immune-related AEs (grade ≥3), proportion of subjects requiring treatment discontinuation due to AEs and proportion of subjects requiring treatment discontinuation due to immune-related AEs. Severity of AEs was graded per the National Cancer Institute Common Terminology Criteria for Adverse Events version 4.03.

PK parameters included C_av_, AUC_0-t_, C_max_, AUC_0-∞_, T_1/2_, K_el_, Cl, C_trough_ after a single dose; AUC_τ,ss,_ C_av,ss_, C_max,ss_, C_trough,ss_ after multiple doses.

The protocol and amendment for this study were reviewed by the ethics committee. The study was conducted in accordance with the principles of Declaration of Helsinki and the Good Clinical Practice guidelines. All the patients provided written informed consent before enrollment.

### Assessment

Tumor size assessment was performed by computed tomography/magnetic resonance imaging (CT/MRI) at screening, on study days 57, 113, and 169, regardless of suspension of the study therapy. Tumor response was assessed per RECIST 1.1 and irRECIST and all efficacy endpoints were based on assessments performed by blinded independent central review (BICR) committee.

Safety analyses included vital sign assessment, physical examination, electrocardiograms, echocardiogram, collection of blood samples for serum chemistry, hematology, coagulation, and thyroid function tests, urinalysis, as well as adverse event assessments. AEs were graded with the use of the National Cancer Institute Common Terminology Criteria for Adverse Events version 4.03.

The schedule of visits and procedures in the FLAT study corresponded to that of the MIRACULUM study.

### Statistical analysis

For the FLAT study, the clinical non-inferiority for ORR of Prolgo 250 mg Q3W versus Prolgo 1 mg/kg Q2W was defined using an 80% retention of the ORR from the confirmatory part of the MIRACULUM study. In the confirmatory part of the MIRACULUM study, ORR per RECIST 1.1 of 20/58 (34.48%) in the per protocol (PP) population and 20/61 (32.79%) in the modified Intention-to-Treat (mITT) population were observed. The clinical relevance of the 80% retention of ORR was justified based on available data from the MIRACULUM study and other studies of prolgolimab and the previous standard reference therapy, dacarbazine, in patients with advanced melanoma as first-line therapy (18–26). According to the combined data from these studies, the proportion of patients with an objective response among patients receiving dacarbazine was 8.9%; the proportion of patients with an objective response among patients receiving prolgolimab was 34.9%. The lower bound of the one-sided 95% confidence interval (CI) for the difference in the proportions of responders was 20.15%.

The FLAT study required a minimum of 91 subjects to demonstrate non-inferiority using a non-inferiority margin of −0.2 for the lower limit of the CI for the risk difference with an 80% power and a one-sided significance level of 0.05 (using the PP population’s response rate from the confirmatory part of the MIRACULUM study as the expected ORR in the sample size calculation). Taking into account the possible dropout rate of 17%, at least 110 subjects were planned to be included in the study.

Statistical analysis was performed using SAS version 9.4 (SAS Institute Inc, Cary, NC). A primary analysis was conducted using PROC GENMOD, which included fitting a model under the null hypothesis. This model specified a binomial distribution with an identity link, incorporating an intercept parameter and a parameter for the strata “AJCC 8 metastatic stage at the time of screening (M1c or M1d vs. other stages).” This stratification factor was determined based on the exploratory analysis of data from the MIRACULUM study. An offset of zero was set for the control group, and an offset equal to the non-inferiority margin was set for the test group. In contrast, the model fitted under the alternative hypothesis included parameters for treatment and strata, with no offset values specified. A one-sided stratified 95% CI for the risk difference, defined as difference in the proportion of subjects with an objective response between the compared treatment regimens, was constructed using the treatment parameter estimate from the model fit under the alternative hypothesis. The non-inferiority *p*-value was computed using the Deviance statistic, which represents twice the difference in log-likelihood values between the two models. Missing data for the primary endpoint, due to any reason, were accounted for in the analysis using non-responder imputation.

The main efficacy and safety analyses were performed in the mITT population (patients who received at least one dose of Prolgo). The missing tumor response evaluations were considered a sign of insufficient response to therapy. Patients without assessments were considered non-responders and conservatively included in denominators. The following rules were used for missing and not evaluable (NE) tumor response assessments (per RECIST 1.1): when no imaging was done at a particular time point, the patient was NE at that time point. When assessing the Best Overall Response (BOR), if a subject had missing tumor response assessments at all visits or was classified as NE for the BOR according to RECIST 1.1, then the subject was considered a non-responder. The single-dose PK population included all patients who received an infusion of Prolgo and for whom not more than three post-dose 1 blood samples were unavailable. The multiple-dose PK population included all patients for whom not more than three post-dose blood samples after the fifth administration were missing. The C_trough_ population included patients who received at least 1 dose of Prolgo and for whom at least 1 pre-dose 1 concentration and at least 1 concentration before any dose were available. The immunogenicity population included all enrolled subjects who received at least one dose of the study drug, with evaluable serum samples collected prior to the administration of the study drug and at least at one subsequent visit.

## Results

### Study population

**Figure 1:**
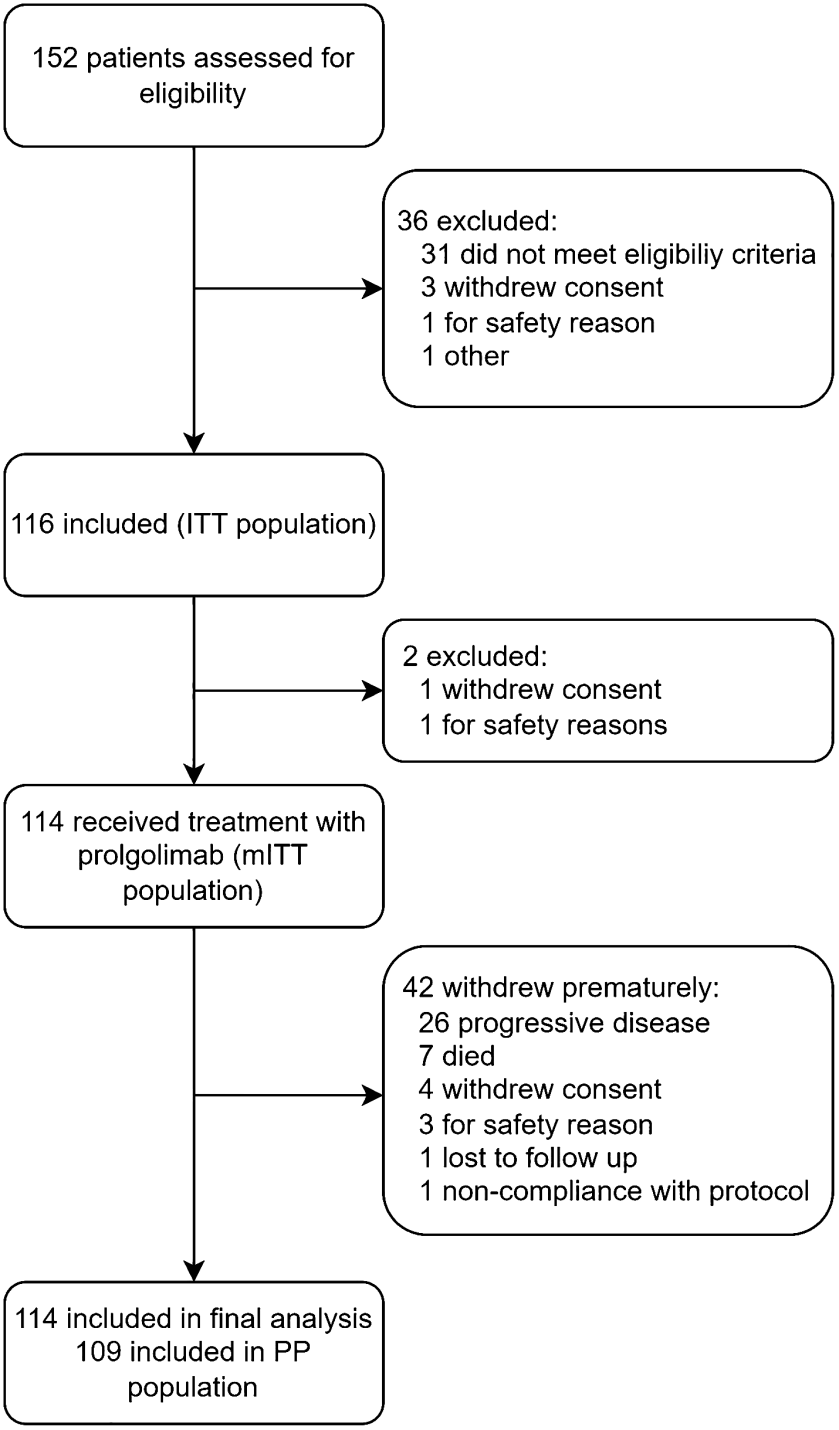
FLAT flowchart.

FLAT was initiated on 13 May 2022; 114 patients from 14 sites across Russia were treated with Prolgo 250 mg Q3W (Prolgo 250 mg group, **Figure 1**). Sixty-one patients from MIRACULUM who received Prolgo 1 mg/kg Q2W comprised a control group (mITT population in the phase 3 part, Prolgo 1 mg/kg group). Baseline demographics and clinical characteristics of patients were generally well balanced and presented in **Table 1**. All patients in both groups received Prolgo as first-line treatment. The number of subjects with non-cutaneous melanoma (mucosal or uveal melanoma) in the Prolgo 250 mg group was 4/114 (3.5%), in the Prolgo 1 mg/kg group—1/61 (1.6%).

**Table 1:** Demographic and disease characteristics of the patients at baseline (mITT population).

Parameter	Prolgo 250 mg (*N* = 114)	Prolgo 1 mg/kg (*N* = 61)
Median age (range), years	63 (32–89)	58 (29–83)
Sex, *n* (%)		
Male	53 (46.5)	24 (39.3)
Female	61 (53.5)	37 (60.7)
Median weight (range), kg	80 (43–180)	74 (51–130)
Metastasis stage, AJCC classification 8, *n* (%)		
M0^1^	6 (5.3)	NA
M1^2^	2 (1.8)	NA
M1a	37 (32.5)	11 (18.0)
M1b	17 (14.9)	17 (27.9)
M1c	39 (34.2)	20 (32.8)
M1d	13 (11.4)	13 (21.3)
Brain metastasis, *n* (%)	12 (10.5)	13 (21.3)
Elevated baseline LDH level, *n* (%)	26 (22.8)	18 (29.5)
Tumor volume at screening, *n* (%)^3^		
≤ 100 mm	95 (83.3)	44 (72.1)
> 100 mm	19 (16.7)	17 (27.9)
BRAF status, *n* (%)		
BRAF wild type	48 (42.1)	9 (14.8)
BRAF mutation	31 (27.2)	18 (29.5)
Test has not been performed	35 (30.7)	34 (55.7)

^1^Unresectable stage III; ^2^further classification of the metastasis stage was not provided (mucosal melanoma); ^3^sum of diameters of target lesions.

### Efficacy

**Figure 2:**
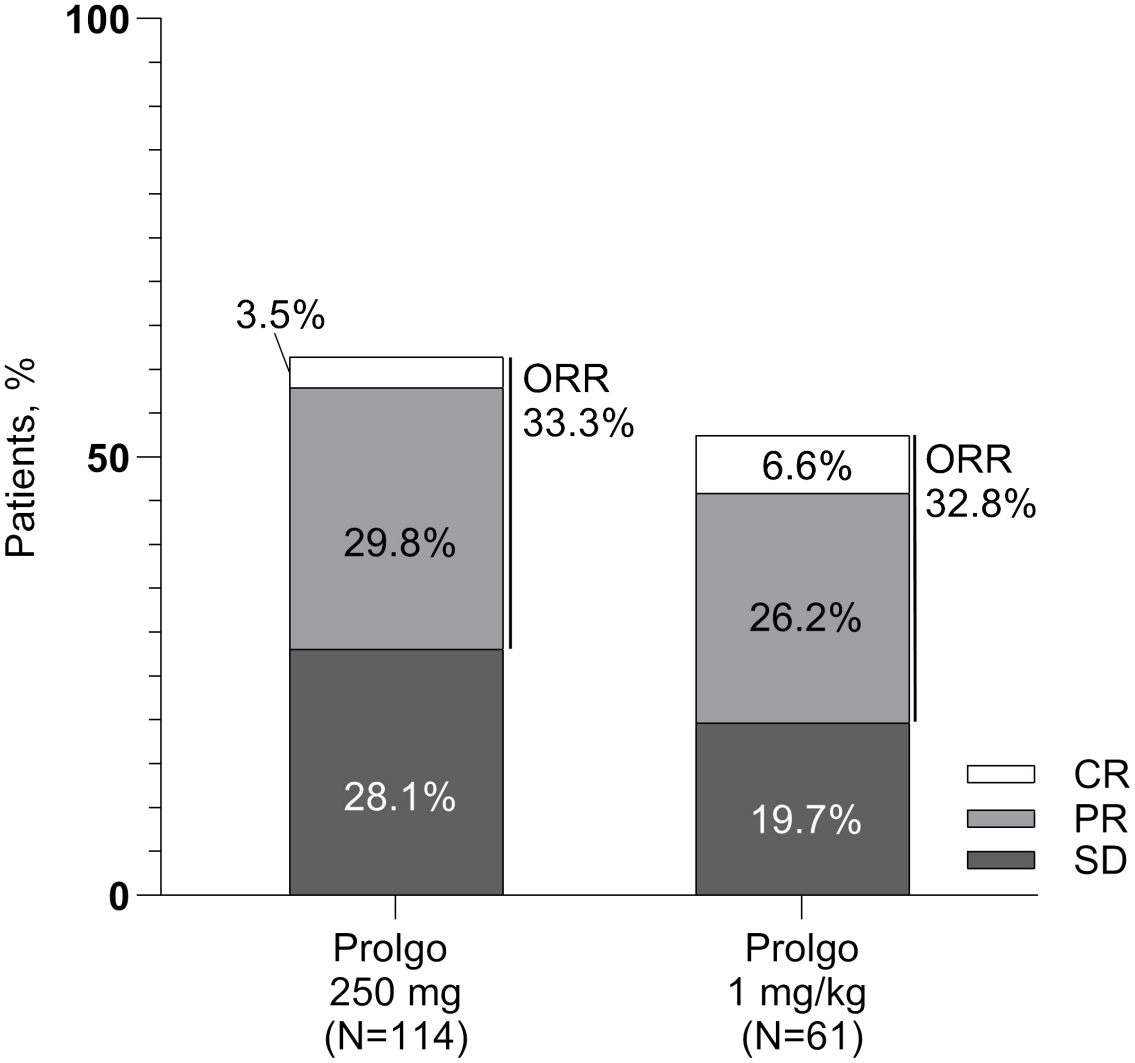
Response rates for patients in the mITT population. ORR, overall response rate; SD, stable disease; PR, partial response; CR, complete response.

On independent central review, 38/114 (33.3%, 95% CI: 24.8–42.8) patients in the Prolgo 250 mg group responded to treatment as per RECIST 1.1 criteria, compared with 20/61 (32.8%, 95% CI: 21.3, 46.0) in the Prolgo 1 mg/kg group (risk difference: 0.00, 95% CI: −0.12 to NA, *p* = 0.0082, log likelihood function; **Table 2**; **Figure 2**), meeting the non-inferiority criterion. ORR results were consistent across prespecified subgroups (**Supplementary Figure S1**).

**Table 2:** Best response according to RECIST 1.1 criteria in the mITT population, by independent central review.

	Prolgo 250 mg (*N* = 114)	Prolgo 1 mg/kg (*N* = 61)
Objective response, *n* (%) (95% CI^1^)	38 (33.3) (24.8; 42.8)	20 (32.8) (21.3; 46.0)
Disease control, *n* (%) (95% CI^1^)	70 (61.4) (51.8; 70.4)	32 (52.5) (39.3; 65.4)
Best overall response, *n* (%)		
Complete response	4 (3.5)	4 (6.6)
Partial response	34 (29.8)	16 (26.2)
Stable disease	32 (28.1)	12 (19.7)
Progressive disease	33 (28.9)	26 (42.6)
Not evaluable	11 (9.6)	3 (4.9)
Median time to objective response, months (95% CI)^2^	1.906 (1.873; 2.103)	2.070 (1.873; 3.680)
Median duration of response, months (95% CI)^2,3^	NA (3.745; NA)	9.429 (4.862; NA)

^1^CI by exact Clopper-Pearson method. ^2^Kaplan–Meier estimate, N = 38 for the Prolgo 250 mg group, N = 20 for the Prolgo 1 mg/kg group. ^3^Number of subjects with event: n = 5 for the Prolgo 250 mg group, n = 6 for the Prolgo 1 mg/kg group. NA, not applicable.

**Figure 3:**
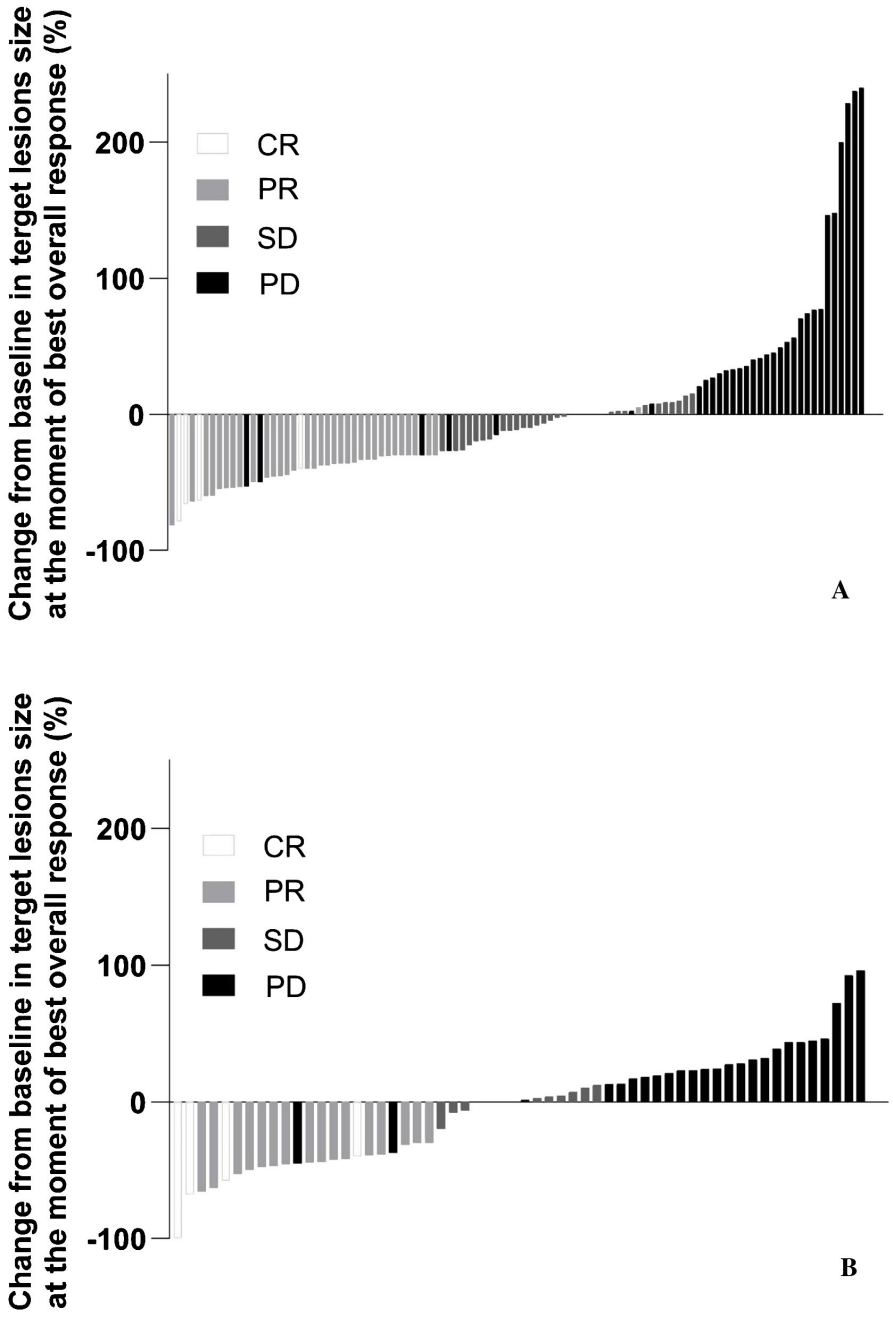
Change from baseline in target lesions size at the moment of best overall response. mITT population. **(A)** Results from the FLAT study; **(B)** Results from the MIRACULUM study. PD, progressive disease; SD, stable disease; PR, partial response; CR, complete response; NE, not evaluation.

The median time to objective response was similar between the Prolgo 250 mg group (1.906, 95% CI: 1.873–2.103) and the Prolgo 1 mg/kg group (2.070, 95% CI: 1.873–3.680). The median duration of response was not reached for the Prolgo 250 mg group (95% CI: 3.745–NA) and was 9.429 months (95% CI: 4.862–NA) for the Prolgo 1 mg/kg group. Depth of response is demonstrated as change from baseline in target lesions size at the moment of best overall response (**Figure 3**).

### Pharmacokinetics and immunogenicity

**Figure 4:**
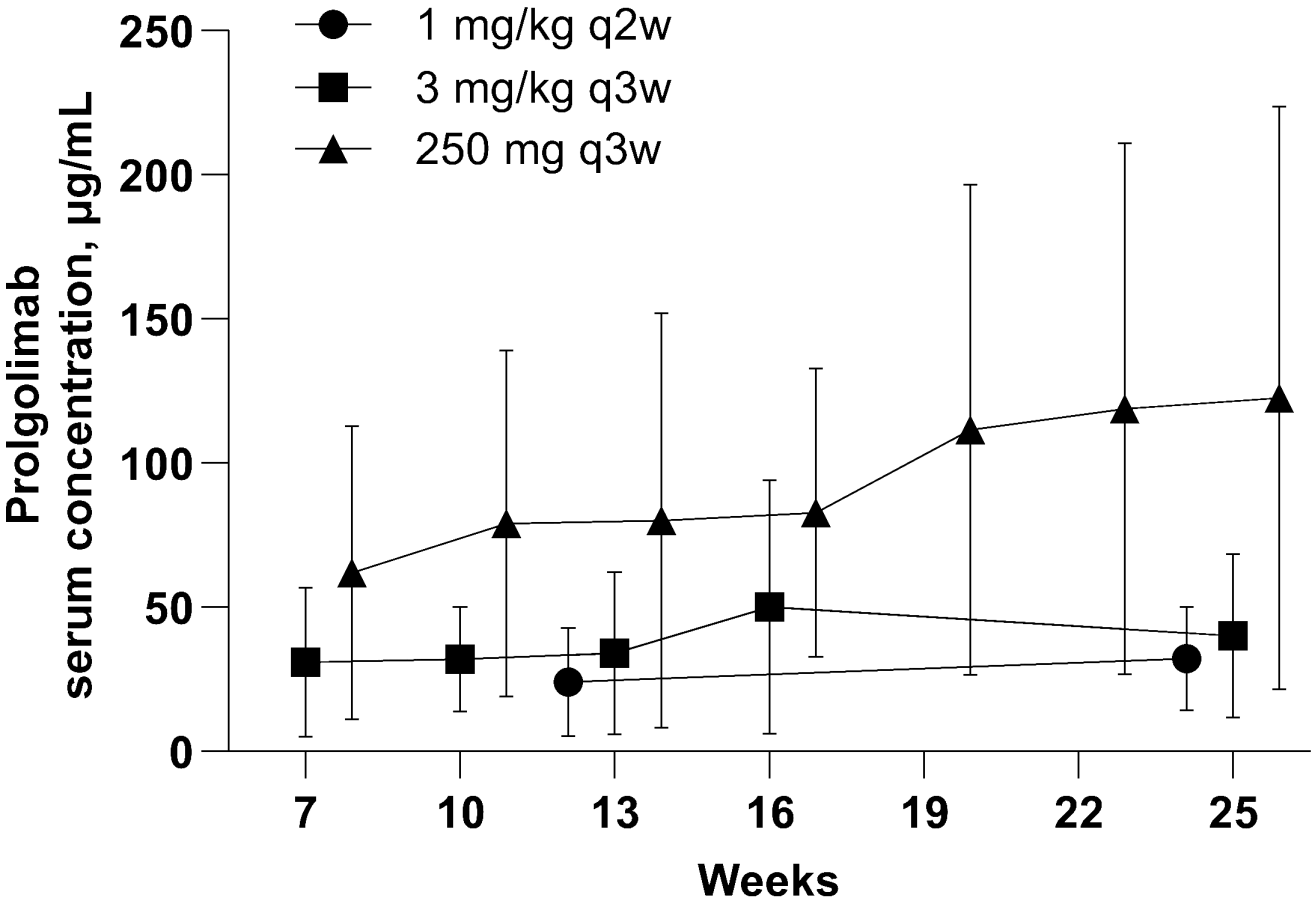
Prolgolimab trough concentraion (mcg/ml). Population for the PK analysis of C_trough._ The straight middle line represents the mean and the whiskers represent standard deviation.

In this analysis, PK data obtained with flat dosing regimen were compared with those obtained with two weight-based dosing regimens in the MIRACULUM study and not previously published (1 mg/kg Q2W and 3 mg/kg Q3W). **Figure 4** illustrates that C_trough_ values with flat dosing were consistently higher to weight-based dosing in the overall C_trough_ population. Flat dosing had adequate exposure for all bodyweight subgroups, as geometric mean and maximum C_trough_ with Prolgo 250 mg were higher than that with Prolgo 1 mg/kg Q2W and 3 mg/kg Q3W (**Supplementary Table S1**). Other PK parameters are also presented in the **Supplementary Tables**. However, since PK data were obtained from different studies conducted in different years with slight modifications of bioanalytical method and different reference standards used, caution should be taken when comparing PK data across these studies.

In the patients evaluable for immunogenicity in the FLAT study (*N* = 95), no one was positive for anti-Prolgo binding antibodies.

### Safety

Flat dosing regimen of Prolgo was well tolerated: there were no treatment-related deaths, AEs of any grade were reported in 85/114 (74.6%) patients, 21/114 (18.4%) patients experienced at least one AE grade ≥3, 60 of 114 patients (52.6%) had at least one treatment-related AE. The safety profiles of Prolgo 250 mg Q3W and Prolgo 1 mg/kg Q2W were similar (**Table 3**). Treatment-related AEs occurred at a similar frequency across flat and weight-based regimens, but the incidence of immune-related AEs was lower in the Prolgo 250 mg group. Treatment discontinuation as a result of AE(s) was needed in 2/114 (1.8%) patients in the Prolgo 250 mg group and in 3/61 (4.9%) patients in the Prolgo 1 mg/kg group. The frequency and profile of AE were comparable in both groups (**Supplementary Tables S2**, **S3**). The most common AEs (>5% of patients in either group) are presented in the appendix.

**Table 3:** Safety parameters: mITT population.

Parameter	Prolgo 250 mg (*N* = 114) *n* (%)	Prolgo 1 mg/kg (*N* = 61) *n* (%)
Proportion of subjects with adverse events	85 (74.6)	47 (77.0)
Proportion of subjects with severe adverse events (grade ≥ 3)	21 (18.4)	14 (23.0)
Proportion of subjects with serious adverse events	6 (5.3)	6 (9.8)
Proportion of subjects with any СТСАЕ grades adverse reactions	60 (52.6)	34 (55.7)
Proportion of subjects with immune-related adverse events	25 (21.9)	20 (32.8)
Proportion of subjects with severe immune-related adverse events (grade ≥ 3)	0	4 (6.6)
Proportion of subjects requiring discontinuation of study drug due to adverse events	2 (1.8)	3 (4.9)
Proportion of subjects requiring discontinuation of study drug due to immune-related adverse events	0	1 (1.6)

There was no clinically meaningful difference in the overall tolerability and safety profiles between weight subgroups (≤65 kg, >65 kg, and <85 kg, ≥85 kg) and C_trough, ss_ subgroups (≥mean, <mean) in the FLAT study (**Supplementary Tables S6**, **S7**).

## Discussion

This study recruited patients with unresectable or metastatic melanoma who have not previously received therapy for unresectable or metastatic disease, as well as those who have not received therapy with anti-CTLA4 and/or anti–PD-1/PD-L1/PD-L2 or targeted therapies. The population of subjects in this study is selected in such a way that it fully corresponds to stage 3 of study BCD-100-2/MIRACULUM (historical control). The use of historical control to demonstrate non-inferiority is driven by the ability to objectively assess and control parameters that influence the number of subjects with response to therapy and are independent of the efficacy of the drug itself. Such factors are patient population determined by eligibility criteria, prior and concomitant therapy, methods and timing of efficacy assessment, study sites, as well as parameters such as baseline tumor size, performance status, concomitant disorders, and presence of CNS metastasis.

ORR has been chosen as a primary endpoint for this non-inferiority study since it represents direct clinical benefit. It is a measure of drug anti-tumor activity and is less susceptible to the influence of disease assessment schedules or patients dropping out before disease progression. Risk factors affecting the development of objective response to prolgolimab therapy in patients with unresectable or metastatic melanoma can be determined based on the results of already completed clinical study MIRACULUM, and these factors can be considered in statistical analysis of the data when comparing the treatment efficacy based on the results of this study with treatment efficacy in MIRACULUM study. Thus, although this study involved the use of historical control, the availability of the results of the MIRACULUM study in combination with the objectivity of the primary endpoint assessment allows an objective comparison of the efficacy of two treatment regimens of prolgolimab: 250 mg Q3W compared with 1 mg/kg Q2W.

For the same purpose, FLAT study was conducted in only those study sites that participated in the MIRACULUM study. The eligibility criteria in FLAT study were the same to MIRACULUM study. Therefore, this allowed using the results of study BCD-100-2/MIRACULUM as a historical control to demonstrate the non-inferiority of prolgolimab 250 mg Q3W versus 1 mg/kg Q2W in terms of overall response.

The population of phase 3 part of the MIRACULUM study included 1/61 (1.6%) patient with atypical melanoma (mucosal or uveal melanoma). Patients with atypical melanoma are less sensitive to anti–PD-1 drugs, such as prolgolimab. In this regard, to ensure the adequacy of comparison of the regimens of prolgolimab 1 mg/kg Q2W and 250 mg Q3W in study BCD-100-8/FLAT, the enrollment of patients with atypical melanoma was limited to ensure a comparable number of such subjects: 4/114 patients (3.5%).

Based on the above, it can be concluded that the FLAT study design allows for an objective comparison of the efficacy of two treatment regimens with prolgolimab.

The Q3W flat dose was selected based on population pharmacokinetics modeling along with efficacy and safety data on weight-based regimens from the MIRACULUM study and as part of the FLAT study the real PK data obtained with flat dosing regimen were compared with those obtained with two weight-based dosing regimens in the MIRACULUM. The results demonstrate that C_trough_ values with flat dosing were consistently higher to weight-based dosing in the overall C_trough_ population, but since PK data were obtained from different studies conducted in different years with slight modifications of bioanalytical method and different reference standards used, caution should be taken when comparing PK data across these studies. The acceptable accuracy and the precision of ELISA should also be taken into consideration.

Anti–PD-1 mAbs with high molecular weights have more complex PK/PD characteristics than standard small molecules. These features should be considered when determining the dosage regimen. They typically have a limited volume of distribution and are thought to be largely confined to the vascular and interstitial spaces. They are primarily eliminated via three mechanisms: a non-specific clearance with pinocytosis by vascular endothelial cells; a specific target-mediated drug disposition caused by the specific Fab region of the antibody-antigen–mediated endocytosis and a non-specific receptor-mediated endocytosis through the Fc domain of the antibody binding with FcγR-expressing cells (27). The neonatal Fc receptor (FcRn) plays an important role in the nonspecific elimination pathway, while the binding affinity of a mAb to the target and the extent of the target expression are significant factors in the target-mediated pathway. Therefore, the mAb’ PK can be affected by various potential covariates including target antigen expression levels, serum protein levels, and disease status, as well as patient demographics such as age, sex, and body size. Consequently, body size may only explain a small portion of the overall inter-individual variability of PK parameters of mAbs. Therefore, the large therapeutic window and the relatively small contribution of body size to the variability in PK and therapeutic outcome may offer more flexibility in the mAb dosing strategy (28).

It should be considered that neither prolgolimab nor other anti–PD-1 antibodies (e.g., pembrolizumab) seem to have direct correlation of efficacy and safety parameters with the dose of the drug, because they saturate target with the doses much lower than approved doses. However, this conclusion requires further confirmation by sufficient amount of clinical data. All anti–PD-1 drugs, which have switched to flat dose use conservative way and justify flat dose comparable to the approved one with the help of pharmacokinetic modelling. For example, pembrolizumab switched from a dose 2 mg/kg to the flat dose 200 mg. The flat dose derived from pharmacokinetic model has maximally predictable efficacy and safety because similar dose was studied in clinical trials. Additionally, there are no data confirming that lower flat dose will result in improved safety or efficacy. JSC BIOCAD conducted pharmacokinetic modelling and showed that the dose 250 mg provides prolgolimab blood concentration no less than the doses of 1 mg/kg and 3 mg/kg, which were studied in pivotal study BCD-100-2/MIRACULUM and demonstrated very similar results in terms of safety and efficacy in patients with advanced melanoma.

The results of the efficacy analysis demonstrate comparable efficacy of flat and weight-based regimens of prolgolimab. The safety analysis shows that the safety profile of the fixed-dose regimen is also consistent with other prolgolimab studies and that there is no clinically meaningful difference in the overall tolerability and safety profiles between weight subgroups and C_trough, ss_ subgroups and between two dosing regimens. The observed lower incidence of irAEs in the 250 mg group might be explained by improved skills of clinicians for management patients receiving immunotherapy considering the elapsed time interval between the two studies (MIRACULUM and FLAT). Therefore, despite the fact that the pharmacokinetics parameters of prolgolimab in 250 mg Q3W is slightly higher than a dose of 1 mg/kg Q2W, this did not affect either the efficacy or safety. Higher pharmacokinetics parameters could also be explained by circulating blood volume increasing with body weight. PD-1 antibody low permeability outside the bloodstream reflects in higher concentrations of the drug in the blood for patients with lower body weight (5). Taking into account that PD-1 receptor saturation has already been achieved (as it occurs at minimal concentrations of anti–PD-1 drugs), there are not negative consequences of increasing the dose, which is affected in comparable efficacy and safety regardless of body weight.

Compared with the approved regimen of prolgolimab 1 mg/kg Q2W and 3 mg/kg Q3W, the fixed dosing has certain advantages: prolgolimab is currently available in 50- or 200-mg vials. When using a weight-based dosing regimen, the contents of the final vial are generally incompletely administered, and the remaining drug product is discarded as per labeling instructions. In usual clinical practice it might potentially be used for another patient, raising quality concerns and, consequently, potential safety concerns as it represents a source of infection when it is used inappropriately outside of the clinical trial setting. Therefore, prolgolimab fixed dose allows enables the efficient utilization of the entire vial of the drug, eliminating the need to discard any unused medication. Additionally, it mitigates the risk of inadvertently administering an incorrect dose due to human error. Rarer prolgolimab administrations are convenient for physicians and patients, reducing the burden on the healthcare system: a rarer dosing regimen reduces the frequency of hospital visits for infusions of the prolgolimab. Thus, when the drug is administered once every 2 weeks, patients have to visit the hospital 26 times a year, and when the drug is administered once every 3 weeks—only 17 times. This, in turn, leads to a reduction in the expenditures of healthcare institutions for the treatment of patients.

Nowadays, prolgolimab dose 250 mg Q3W was approved for use in patients with melanoma and NSCLC in the Russian Federation, and clinical results show similar efficacy and safety in these indications among doses (1 mg/kg Q2W, 250 mg Q3W, and 3 mg/kg Q3W) in the trials supporting these indications (8, 11).

## Data Availability

The original contributions presented in the study are included in the article/**Supplementary Material**. Further inquiries can be directed to the corresponding author.
