# Supplementary material for: A phase III study to access the safety and efficacy of prolgolimab 250 mg fixed dose administered every 3 weeks versus prolgolimab 1 mg/kg every 2 weeks in patients with metastatic melanoma (FLAT)
Source: Front Oncol. 2024 Sep 4;14:1385685. doi: 10.3389/fonc.2024.1385685 (PMC11408354; doi:10.3389/fonc.2024.1385685)
Supplement: Supplementary file 1 [file DataSheet1.docx]

Supplementary Material

## Supplemental Figures Legend

**Figure S1.** **Forest plot for objective response rate per RECIST 1.1 criteria. Subgroup analysis. mITT population**

## Supplemental Tables

Table S1. C_trough_ weeks 13th and 25th in different weight groups descriptive statistics (mcg/ml). Population for the PK analysis of C_trough_

| Group  Statistic | 1 mg/kg q2w  (N=52) | 3 mg/kg q3w  (N=42) | 250 mg q3w  (N=107) |
| --- | --- | --- | --- |
|  |  |  |  |
| C_trough_ week 13 |  |  |  |
|  |  |  |  |
| ≤ 65 kg |  |  |  |
| N | 18 | 6 | 11 |
| Mean | 23.70 | 38.31 | 83.41 |
| Geom. mean | 15.27 | 26.77 | 72.98 |
| Median | 18.03 | 23.00 | 76.69 |
| Minimum | 0.0 | 10.1 | 38.6 |
| Maximum | 74.1 | 114.3 | 159.4 |
| Q1 | 10.69 | 15.35 | 44.54 |
| Q3 | 32.66 | 40.73 | 118.71 |
| SD | 19.12 | 39.29 | 44.92 |
| CV (%) | 80.7 | 102.6 | 53.9 |
|  |  |  |  |
| >65 kg, <85 kg |  |  |  |
| N | 20 | 16 | 47 |
| Mean | 21.76 | 30.29 | 79.09 |
| Geom. mean | 18.91 | 23.80 | 54.51 |
| Median | 19.06 | 26.81 | 53.72 |
| Minimum | 7.2 | 7.6 | 0.0 |
| Maximum | 59.9 | 83.3 | 385.5 |
| Q1 | 12.98 | 14.89 | 38.65 |
| Q3 | 25.58 | 41.16 | 93.58 |
| SD | 12.86 | 21.89 | 71.37 |
| CV (%) | 59.1 | 72.3 | 90.2 |
|  |  |  |  |
| ≥ 85 kg |  |  |  |
| N | 6 | 20 | 36 |
| Mean | 32.47 | 36.06 | 80.19 |
| Geom. mean | 21.22 | 27.88 | 57.28 |
| Median | 17.56 | 28.40 | 55.12 |
| Minimum | 6.8 | 6.4 | 6.8 |
| Maximum | 86.1 | 129.8 | 421.0 |
| Q1 | 10.34 | 14.83 | 34.78 |
| Q3 | 48.50 | 38.00 | 92.75 |
| SD | 32.15 | 30.18 | 82.75 |
| CV (%) | 99.0 | 83.7 | 103.2 |
|  |  |  |  |
| C_trough_ week 25 |  |  |  |
|  |  |  |  |
| ≤ 65 kg |  |  |  |
| N | 12 | 2 | 9 |
| Mean | 36.40 | 54.59 | 117.15 |
| Geom. mean | 33.47 | 52.58 | 86.92 |
| Median | 42.39 | 54.59 | 62.06 |
| Minimum | 11.4 | 39.9 | 22.6 |
| Maximum | 52.0 | 69.3 | 301.3 |
| Q1 | 24.20 | 47.26 | 52.22 |
| Q3 | 46.82 | 61.92 | 152.77 |
| SD | 13.59 | 20.74 | 95.65 |
| CV (%) | 37.3 | 38.0 | 81.6 |
|  |  |  |  |
| >65 kg, <85 kg |  |  |  |
| N | 13 | 10 | 35 |
| Mean | 26.57 | 27.19 | 121.30 |
| Geom. mean | 16.69 | 20.11 | 86.84 |
| Median | 19.13 | 19.62 | 95.21 |
| Minimum | 0.0 | 4.6 | 8.1 |
| Maximum | 64.5 | 57.9 | 444.3 |
| Q1 | 13.63 | 11.82 | 46.77 |
| Q3 | 39.35 | 44.69 | 158.30 |
| SD | 20.03 | 19.47 | 97.60 |
| CV (%) | 75.4 | 71.6 | 80.5 |
|  |  |  |  |
| ≥ 85 kg |  |  |  |
| N | 3 | 13 | 30 |
| Mean | 38.90 | 49.42 | 125.67 |
| Geom. mean | 32.14 | 37.82 | 89.19 |
| Median | 46.12 | 51.98 | 82.19 |
| Minimum | 12.4 | 7.3 | 16.0 |
| Maximum | 58.2 | 111.1 | 374.4 |
| Q1 | 29.25 | 23.91 | 48.45 |
| Q3 | 52.17 | 72.08 | 164.52 |
| SD | 23.76 | 32.02 | 110.28 |
| CV (%) | 61.1 | 64.8 | 87.7 |
|  |  |  |  |

Table S2. Prolgolimab PK parameters after the first infusion descriptive statistics. Population for the PK analysis after the first infusion

| Parameter  Statistic | 3 mg/kg q3w | 250 mg q3w |
| --- | --- | --- |
|  |  |  |
| AUC_0-t_ (h·mcg/ml) |  |  |
| N | 62 | 113 |
| Mean | 14952.95 | 25603.76 |
| Geom. mean | 12268.46 | 21702.50 |
| Median | 11324.97 | 24211.01 |
| Minimum | 2160.7 | 3597.5 |
| Maximum | 46790.9 | 93781.1 |
| Q1 | 8922.00 | 16843.02 |
| Q3 | 16686.91 | 30439.58 |
| SD | 10190.72 | 14651.18 |
| CV (%) | 68.2 | 57.2 |
|  |  |  |
| AUC_0-∞_ (h·mcg/ml) |  |  |
| N | 35 | 99 |
| Mean | 23256.66 | 47527.30 |
| Geom. mean | 20865.78 | 38287.37 |
| Median | 19970.20 | 40096.79 |
| Minimum | 5230.8 | 4440.9 |
| Maximum | 53191.6 | 203581.9 |
| Q1 | 14857.75 | 28056.04 |
| Q3 | 30061.92 | 57648.25 |
| SD | 11333.79 | 32500.58 |
| CV (%) | 48.7 | 68.4 |
|  |  |  |
| C_max_ (mcg/ml) |  |  |
| N | 62 | 113 |
| Mean | 82.92 | 133.96 |
| Geom. mean | 67.53 | 106.93 |
| Median | 63.46 | 111.41 |
| Minimum | 11.9 | 15.8 |
| Maximum | 269.5 | 639.0 |
| Q1 | 44.99 | 78.22 |
| Q3 | 107.49 | 149.24 |
| SD | 55.08 | 103.35 |
| CV (%) | 66.4 | 77.1 |
|  |  |  |
| C_av_ (mcg/ml) |  |  |
| N | 62 | 113 |
| Mean | 29.67 | 52.09 |
| Geom. mean | 24.34 | 43.77 |
| Median | 22.47 | 48.31 |
| Minimum | 4.3 | 7.1 |
| Maximum | 92.8 | 185.6 |
| Q1 | 17.70 | 33.68 |
| Q3 | 33.11 | 61.57 |
| SD | 20.22 | 30.23 |
| CV (%) | 68.2 | 58.0 |
|  |  |  |
| C_trough_ (mcg/ml) |  |  |
| N | 53 | 109 |
| Mean | 27.45 | 35.99 |
| Geom. mean | 17.33 | 28.28 |
| Median | 13.84 | 30.03 |
| Minimum | 3.1 | 3.0 |
| Maximum | 146.3 | 133.7 |
| Q1 | 10.53 | 20.84 |
| Q3 | 22.72 | 46.88 |
| SD | 34.30 | 24.83 |
| CV (%) | 125.0 | 69.0 |
|  |  |  |
| Cl (ml/h) |  |  |
| N | 62 | 113 |
| Mean | 23.818 | 14.298 |
| Geom. mean | 19.727 | 11.519 |
| Median | 20.770 | 10.330 |
| Minimum | 2.69 | 2.67 |
| Maximum | 95.80 | 69.49 |
| Q1 | 15.278 | 8.210 |
| Q3 | 29.155 | 14.840 |
| SD | 16.128 | 11.769 |
| CV (%) | 67.7 | 82.3 |
|  |  |  |
| T_1/2_ (h) |  |  |
| N | 38 | 99 |
| Mean | 408.046 | 442.646 |
| Geom. mean | 334.709 | 328.134 |
| Median | 329.850 | 334.130 |
| Minimum | 113.08 | 4.25 |
| Maximum | 1800.38 | 3025.27 |
| Q1 | 255.208 | 209.295 |
| Q3 | 433.153 | 532.335 |
| SD | 354.466 | 425.200 |
| CV (%) | 86.9 | 96.1 |
|  |  |  |
| K_el_ (1/h) |  |  |
| N | 38 | 99 |
| Mean | 0.00239 | 0.00416 |
| Geom. mean | 0.00208 | 0.00211 |
| Median | 0.00210 | 0.00210 |
| Minimum | 0.0004 | 0.0002 |
| Maximum | 0.0061 | 0.1630 |
| Q1 | 0.00163 | 0.00130 |
| Q3 | 0.00270 | 0.00335 |
| SD | 0.00126 | 0.01623 |
| CV (%) | 52.9 | 390.3 |
|  |  |  |

Table S3. Prolgolimab PK parameters after the fifth infusion descriptive statistics. Population for the PK analysis after the fifth infusion

| Parameter  Statistic | 3 mg/kg q3w | 250 mg q3w |
| --- | --- | --- |
|  |  |  |
| AUC_τ,ss_ (h·mcg/ml) |  |  |
| N | 42 | 94 |
| Mean | 24769.00 | 54600.45 |
| Geom. mean | 20630.65 | 44509.23 |
| Median | 22822.81 | 48265.95 |
| Minimum | 5019.6 | 2353.9 |
| Maximum | 82774.6 | 165165.0 |
| Q1 | 11348.84 | 32696.18 |
| Q3 | 31605.63 | 69675.61 |
| SD | 15444.96 | 32052.62 |
| CV (%) | 62.4 | 58.7 |
|  |  |  |
| C_max,ss_ (mcg/ml) |  |  |
| N | 42 | 94 |
| Mean | 124.42 | 237.09 |
| Geom. mean | 105.89 | 208.99 |
| Median | 105.16 | 200.11 |
| Minimum | 36.0 | 47.9 |
| Maximum | 393.0 | 539.1 |
| Q1 | 71.72 | 149.97 |
| Q3 | 154.43 | 317.38 |
| SD | 76.37 | 117.98 |
| CV (%) | 61.4 | 49.8 |
|  |  |  |
| C_av,ss_ (mcg/ml) |  |  |
| N | 42 | 94 |
| Mean | 49.05 | 111.34 |
| Geom. mean | 40.85 | 96.19 |
| Median | 45.19 | 95.85 |
| Minimum | 9.9 | 15.3 |
| Maximum | 163.9 | 327.6 |
| Q1 | 22.47 | 68.46 |
| Q3 | 62.59 | 136.00 |
| SD | 30.58 | 62.11 |
| CV (%) | 62.4 | 55.8 |
|  |  |  |
| C_trough,ss_ (mcg/ml) |  |  |
| N | 30 | 83 |
| Mean | 50.69 | 82.90 |
| Geom. mean | 37.04 | 67.29 |
| Median | 35.36 | 69.56 |
| Minimum | 8.3 | 4.6 |
| Maximum | 176.1 | 216.9 |
| Q1 | 18.86 | 43.91 |
| Q3 | 57.66 | 112.07 |
| SD | 43.98 | 50.97 |
| CV (%) | 86.8 | 61.5 |
|  |  |  |

Table S4. Proportion of Subjects with AEs of Any Severity Reported in More Than 5% of Patients in the Prolgo 250 mg Group. mITT population

| **SOC**  **PT**  **Grade** | **Prolgo 250 mg** **(N=114)** **n (%)** |
| --- | --- |
|  | |
| **Investigations** | 40 (35.1) |
| Grade 1 | 18 (15.8) |
| Grade 2 | 16 (14.0) |
| Grade 3 | 5 (4.4) |
| Grade 4 | 1 (0.9) |
|  | |
| Aspartate aminotransferase increased | 14 (12.3) |
| Grade 1 | 8 (7.0) |
| Grade 2 | 5 (4.4) |
| Grade 4 | 1 (0.9) |
|  | |
| Alanine aminotransferase increased | 13 (11.4) |
| Grade 1 | 6 (5.3) |
| Grade 2 | 4 (3.5) |
| Grade 3 | 3 (2.6) |
|  | |
| Blood alkaline phosphatase increased | 12 (10.5) |
| Grade 1 | 9 (7.9) |
| Grade 2 | 2 (1.8) |
| Grade 3 | 1 (0.9) |
|  | |
| Amylase increased | 10 (8.8) |
| Grade 1 | 5 (4.4) |
| Grade 2 | 4 (3.5) |
| Grade 3 | 1 (0.9) |
|  | |
| Blood urea increased | 10 (8.8) |
| Grade 1 | 10 (8.8) |
|  | |
| Weight loss | 10 (8.8) |
| Grade 1 | 7 (6.1) |
| Grade 2 | 3 (2.6) |
|  | |
| Blood lactate dehydrogenase increased | 8 (7.0) |
| Grade 1 | 8 (7.0) |
|  | |
| Blood creatinine increased | 6 (5.3) |
| Grade 1 | 5 (4.4) |
| Grade 2 | 1 (0.9) |
|  |  |
| Blood uric acid increased | 6 (5.3) |
| Grade 1 | 6 (5.3) |
|  | |
| **Blood and lymphatic system disorders** | 33 (28.9) |
| Grade 1 | 9 (7.9) |
| Grade 2 | 20 (17.5) |
| Grade 3 | 3 (2.6) |
| Grade 4 | 1 (0.9) |
|  | |
| Anemia | 17 (14.9) |
| Grade 1 | 5 (4.4) |
| Grade 2 | 11 (9.6) |
| Grade 3 | 1 (0.9) |
|  | |
| Lymphopenia | 8 (7.0) |
| Grade 1 | 1 (0.9) |
| Grade 2 | 6 (5.3) |
| Grade 3 | 1 (0.9) |
|  | |
| Thrombocytopenia | 6 (5.3) |
| Grade 1 | 4 (3.5) |
| Grade 2 | 1 (0.9) |
| Grade 3 | 1 (0.9) |
|  | |
| **Metabolism and nutrition disorders** | 25 (21.9) |
| Grade 1 | 15 (13.2) |
| Grade 2 | 8 (7.0) |
| Grade 3 | 1 (0.9) |
| Grade 4 | 1 (0.9) |
|  | |
| Hyperglycemia | 12 (10.5) |
| Grade 1 | 7 (6.1) |
| Grade 2 | 4 (3.5) |
| Grade 3 | 1 (0.9) |
|  | |
| Decreased appetite | 12 (10.5) |
| Grade 1 | 9 (7.9) |
| Grade 2 | 3 (2.6) |
|  | |
| **Endocrine disorders** | 25 (21.9) |
| Grade 1 | 9 (7.9) |
| Grade 2 | 16 (14.0) |
|  | |
| Hypothyroidism | 7 (6.1) |
| Grade 1 | 3 (2.6) |
| Grade 2 | 4 (3.5) |
|  | |
| **General disorders and administration site conditions** | 16 (14.0) |
| Grade 1 | 5 (4.4) |
| Grade 2 | 4 (3.5) |
| Grade 3 | 6 (5.3) |
| Grade 5 | 1 (0.9) |
|  | |
| Asthenia | 13 (11.4) |
| Grade 1 | 4 (3.5) |
| Grade 2 | 3 (2.6) |
| Grade 3 | 6 (5.3) |
|  | |
| **Cardiac disorders** | 12 (10.5) |
| Grade 1 | 11 (9.6) |
| Grade 2 | 1 (0.9) |
|  | |
| **Infections and infestations** | 11 (9.6) |
| Grade 1 | 4 (3.5) |
| Grade 2 | 6 (5.3) |
| Grade 3 | 1 (0.9) |
|  | |
| **Gastrointestinal disorders** | 10 (8.8) |
| Grade 1 | 5 (4.4) |
| Grade 2 | 3 (2.6) |
| Grade 3 | 1 (0.9) |
| Grade 4 | 1 (0.9) |
|  |  |
| **Skin and subcutaneous tissue disorders** | 9 (7.9) |
| Grade 1 | 4 (3.5) |
| Grade 2 | 5 (4.4) |
|  | |
| **Musculoskeletal and connective tissue disorders** | 7 (6.1) |
| Grade 1 | 3 (2.6) |
| Grade 2 | 3 (2.6) |
| Grade 3 | 1 (0.9) |
|  | |
| **Nervous system disorders** | 7 (6.1) |
| Grade 1 | 3 (2.6) |
| Grade 2 | 3 (2.6) |
| Grade 5 | 1 (0.9) |
|  | |
| Dizziness | 6 (5.3) |
| Grade 1 | 5 (4.4) |
| Grade 2 | 1 (0.9) |
|  | |
| **Note:** PT, Preferred Term, SOC, System Organ Class. PT and SOC are encoded using MedDRA version 25.1. | |

**Table S5. Proportion of Subjects with AEs of Any Severity Reported in More Than 5% of Patients in the Prolgo 1 mg/kg Group. mITT population**

| **SOC**  **PT**  **Grade** | **BCD-100 1 mg/kg** **(n = 61)** **n (%)** |
| --- | --- |
|  |  |
| **Investigations** | 15 (24.59) |
| Grade 1 | 12 (19.67) |
| Grade 2 | 4 (6.56) |
|  |  |
| Body temperature increased | 5 (8.20) |
| Grade 1 | 3 (4.92) |
| Grade 2 | 2 (3.28) |
|  |  |
| **Blood and lymphatic system disorders** | 12 (19.67) |
| Grade 1 | 6 (9.84) |
| Grade 2 | 5 (8.20) |
| Grade 3 | 1 (1.64) |
|  |  |
| Anemia | 7 (11.48) |
| Grade 1 | 3 (4.92) |
| Grade 2 | 4 (6.56) |
|  |  |
| **Endocrine disorders** | 9 (14.75) |
| Grade 1 | 5 (8.20) |
| Grade 2 | 4 (6.56) |
|  |  |
| Hyperthyroidism | 7 (11.48) |
| Grade 1 | 4 (6.56) |
| Grade 2 | 3 (4.92) |
|  |  |
| **Vascular disorders** | 7 (11.48) |
| Grade 2 | 5 (8.20) |
| Grade 3 | 2 (3.28) |
|  |  |
| Hypertension | 4 (6.56) |
| Grade 2 | 2 (3.28) |
| Grade 3 | 2 (3.28) |
|  |  |
| **General disorders and administration site conditions** | 6 (9.84) |
| Grade 1 | 4 (6.56) |
| Grade 2 | 1 (1.64) |
| Grade 3 | 1 (1.64) |
| Grade 5 | 1 (1.64) |
|  |  |
| Asthenia | 4 (6.56) |
| Grade 1 | 3 (4.92) |
| Grade 3 | 1. (1.64) |

**Table S6. Safety parameters. mITT population, subjects with measured Ctrough after fifth study drug administration in the FLAT study**

| **Parameter** | **Ctrough ≥ Mean (N = 34) n (%)** | **Ctrough < Mean (N = 49) n (%)** |
| --- | --- | --- |
| Proportion of subjects with adverse events | 23 (67.6) | 37 (75.5) |
| Proportion of subjects with severe adverse events (grades 3 or more according СТСАЕ v.4.03) | 5 (14.7) | 6 (12.2) |
| Proportion of subjects with serious adverse events | 0 | 2 (4.1) |
| Proportion of subjects with any СТСАЕ grades adverse reactions | 19 (55.9) | 26 (53.1) |
| Proportion of subjects with immune-related adverse events | 11 (32.4) | 11 (22.4) |
| Proportion of subjects with severe immune-related adverse events (grades 3 or more according СТСАЕ v.4.03) | 0 | 0 |
| Proportion of subjects requiring discontinuation of study drug due to adverse events | 1 (2.9) | 0 |
| Proportion of subjects requiring discontinuation of study drug due to immune-related adverse events | 0 | 0 |
| **Note:** Mean = 82.9 mcg/ml. | | |

**Table S7. Safety parameters. mITT population in the FLAT study**

| **Parameter** | **≤ 65 kg (N = 18) n (%)** | **> 65 - < 85 kg (N = 49) n (%)** | **≥ 85 kg (N = 47) n (%)** |
| --- | --- | --- | --- |
| Proportion of subjects with adverse events | 12 (66.7) | 38 (77.6) | 35 (74.5) |
| Proportion of subjects with severe adverse events (grades 3 or more according СТСАЕ v.4.03) | 3 (16.7) | 9 (18.4) | 9 (19.1) |
| Proportion of subjects with serious adverse events | 1 (5.6) | 3 (6.1) | 2 (4.3) |
| Proportion of subjects with any СТСАЕ grades adverse reactions | 10 (55.6) | 26 (53.1) | 24 (51.1) |
| Proportion of subjects with immune-related adverse events | 2 (11.1) | 11 (22.4) | 12 (25.5) |
| Proportion of subjects with severe immune-related adverse events (grades 3 or more according СТСАЕ v.4.03) | 0 | 0 | 0 |
| Proportion of subjects requiring discontinuation of study drug due to adverse events | 0 | 0 | 2 (4.3) |
| Proportion of subjects requiring discontinuation of study drug due to immune-related adverse events | 0 | 0 | 0 |
