# Supplementary figures and images for: A phase III study to access the safety and efficacy of prolgolimab 250 mg fixed dose administered every 3 weeks versus prolgolimab 1 mg/kg every 2 weeks in patients with metastatic melanoma (FLAT)
Source: Front Oncol. 2024 Sep 4;14:1385685. doi: 10.3389/fonc.2024.1385685 (PMC11408354; doi:10.3389/fonc.2024.1385685)

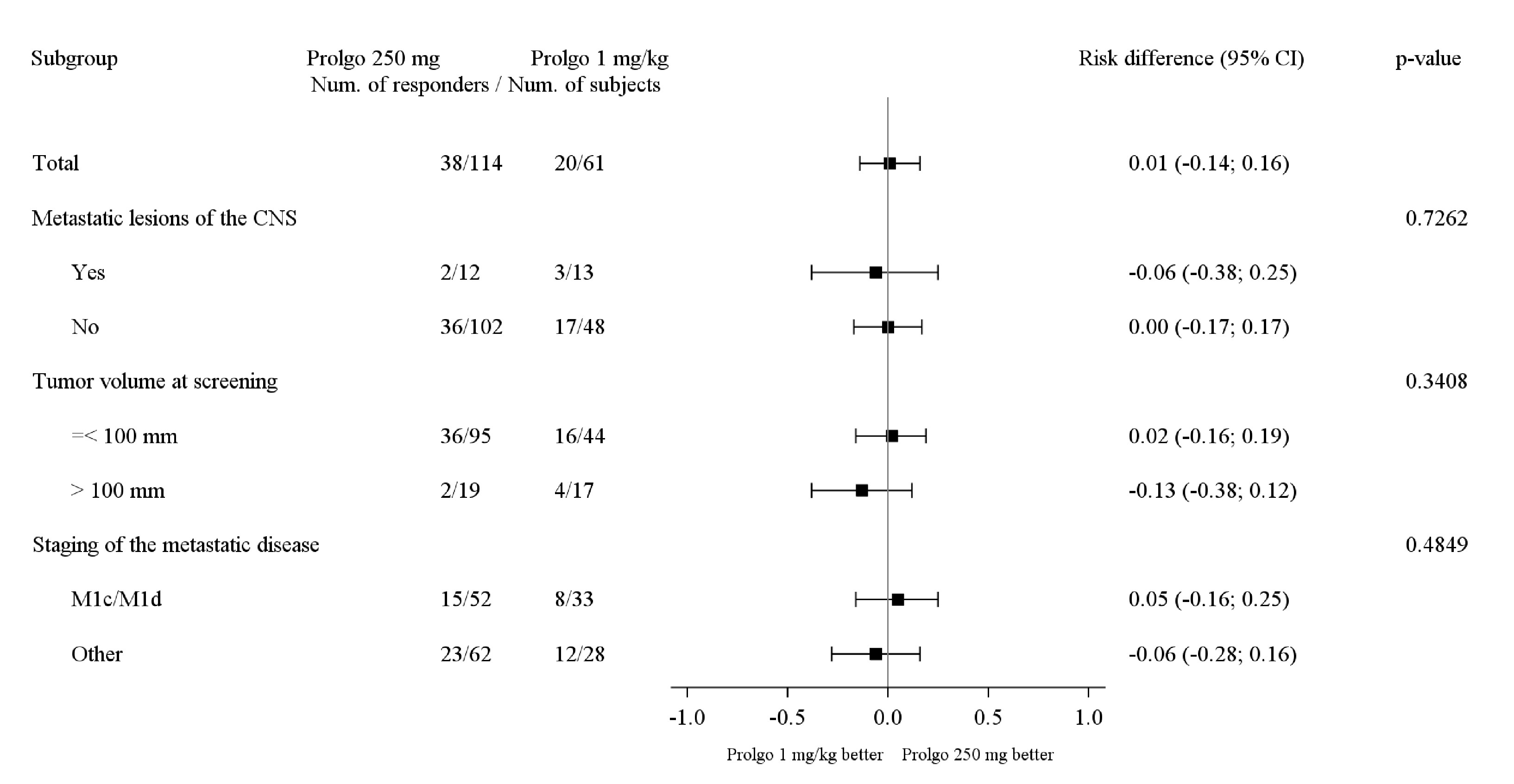

Supplement: Supplementary file 2 [file Image1.jpeg]
